# Polysaccharide from *Artocarpus heterophyllus* Lam. (Jackfruit) Pulp Ameliorates Dextran Sodium Sulfate-Induced Enteritis in Rats

**DOI:** 10.3390/ijms25031661

**Published:** 2024-01-29

**Authors:** Yunlong Li, Yuzi Chen, Chuan Li, Gang Wu, Yanfu He, Lehe Tan, Kexue Zhu

**Affiliations:** 1 School of Food Science and Engineering, Hainan University, Haikou 570228, China; 2 Spice and Beverage Research Institute, Chinese Academy of Tropical Agricultural Sciences, Wanning 571533, China; 3 College of Food Science and Technology, Huazhong Agricultural University, Wuhan 430070, China; 4 Key Laboratory of Processing Suitability and Quality Control of the Special Tropical Crops of Hainan Province, Wanning 571533, China; 5 National Center of Important Tropical Crops Engineering and Technology Research, Wanning 571533, China

**Keywords:** *Artocarpus heterophyllus* Lam., polysaccharide, signaling pathway, inflammation, intestinal barrier

## Abstract

A polysaccharide from *Artocarpus heterophyllus* Lam. (jackfruit) pulp (JFP-Ps) is known for its excellent bioactivities. However, its impact on small intestinal barrier function is still largely unexplored. The study aimed to examine the protection effect of JFP-Ps against dextran sodium sulfate-induced enteritis and its underlying mechanism. This research revealed that JFP-Ps mitigated small intestinal tissue damage by reducing the expression of pro-inflammatory cytokines and promoting the expression of the anti-inflammatory cytokine interleukin-10 in the small intestine. JFP-Ps diminished oxidative stress by bolstering the activity of antioxidant enzymes and reducing the concentration of malondialdehyde in the small intestine. In addition, JFP-Ps may restore the mechanical barrier and inhibit intestinal structure damage by augmenting the expression of short-chain fatty acids (SCFAs) receptors (GPR41/43) and up-regulating the expression of tight junction proteins (occludin). In conclusion, JFP-Ps may positively influence intestinal health by relieving oxidative stress in the small intestine, improving mechanical barrier function, activating the SCFA-GPR41/GPR43 axis, and inhibiting TLR4/MAPK pathway activation. The results augment our comprehension of the bioactivities of JFP-Ps, corroborating its great potential as a functional food.

## 1. Introduction

The alimentary canal, specifically the intestine, serves as the most extensive interface between organisms and their surrounding environment, primarily functioning as the principal site for the digestion and absorption of nutrients [1,2]. As the largest immune organ in the body, the intestine provides a crucial barrier for maintaining overall systemic health [3]. As a physical barrier, the intestine prevents the invasion of foreign antigens, such as microorganisms and toxins [4,5]. The intestinal mucosal barrier plays an important role in the maintenance of intestinal health [6]. Intestinal barrier dysfunction or immune dysregulation can lead to increased intestinal mucosal permeability, which promotes the translocation of intestinal pathogens, further exacerbates intestinal barrier damage, and even induces systemic infection [7]. Therefore, searching for active substances that regulate intestinal barrier function is crucial to human health.

Natural polysaccharides from animals, plants, algae, and microorganisms exhibit favorable bioactivities and have received much attention from researchers due to their low toxic side effects [8,9]. The protective effect of natural polysaccharides against inflammatory bowel disease (IBD) is a hot research topic. Natural polysaccharides have been reported to enhance the integrity of the intestinal epithelial cell mainly through direct or indirect effects, affecting the intestinal immune and biological barriers [4]. Previous studies have shown that polysaccharides extracted from pacific abalone, alfalfa, astragalus, and ginseng maintained intestinal health by regulating the expression of inflammatory cytokines and activating the immune signaling pathway [10,11,12]. Zou et al. [13] found that seaweed-derived polysaccharides improved immune status and intestinal morphology, inhibited oxidative stress, and enhanced the expression of tight junction proteins. Feng et al. [14] discovered that polysaccharides from *yellow sweet potatoes* exerted anti-inflammatory activity by increasing the concentration of short-chain fatty acids (SCFAs) and upregulating the expression of GPR41 receptors.

A polysaccharide purified from the *Artocarpus heterophyllus* Lam. pulp (JFP-Ps) was composed of varied monosaccharides, including glucose, galactose, xylose, rhamnose, arabinose, and galacturonic acid [15]. The immunoregulatory and antitumor properties of JFP-Ps have been reported [16,17]. In our previous study, JFP-Ps was found to possess antioxidant activity, modulate lipid metabolism, and improve the structure of intestinal flora [15,18,19]. However, the protective effects of JFP-Ps on small intestinal injury induced by dextran sodium sulfate (DSS) in rats and its underlying mechanisms need to be further investigated.

## 2. Results

### 2.1. JFP-Ps Alleviated the Damage of Small Intestinal Mucosa

**Figure 1:**
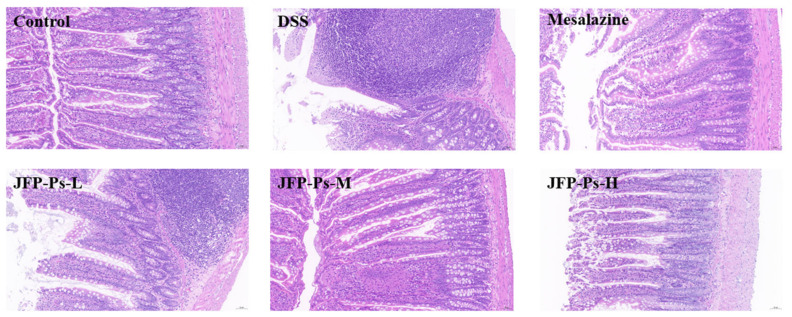
The micromorphology of small intestine (original magnification 200×).

As depicted in Figure 1, the small intestines of rats from the control group showed normal histology, characterized by an unbroken epithelium, properly structured villi and crypts, minimal infiltration of leukocytes, and regular cup cells. In contrast, the DSS-treated rats showed significant histological abnormalities with epithelial erosion, damaged intestinal mucosa, intestinal villi atrophy, and loss of cupped cells. This indicated that DSS disrupted the surface structure of small intestinal tissue. However, JFP-Ps at different doses significantly ameliorated the abnormalities of small intestinal structures, with a relatively tighter arrangement of intestinal villi and higher mucosal layer thickness in the rats. The findings indicated that JFP-Ps effectively preserved the structural integrity of small intestinal mucosa.

### 2.2. JFP-Ps Enhanced the Antioxidant Activities

**Figure 2:**
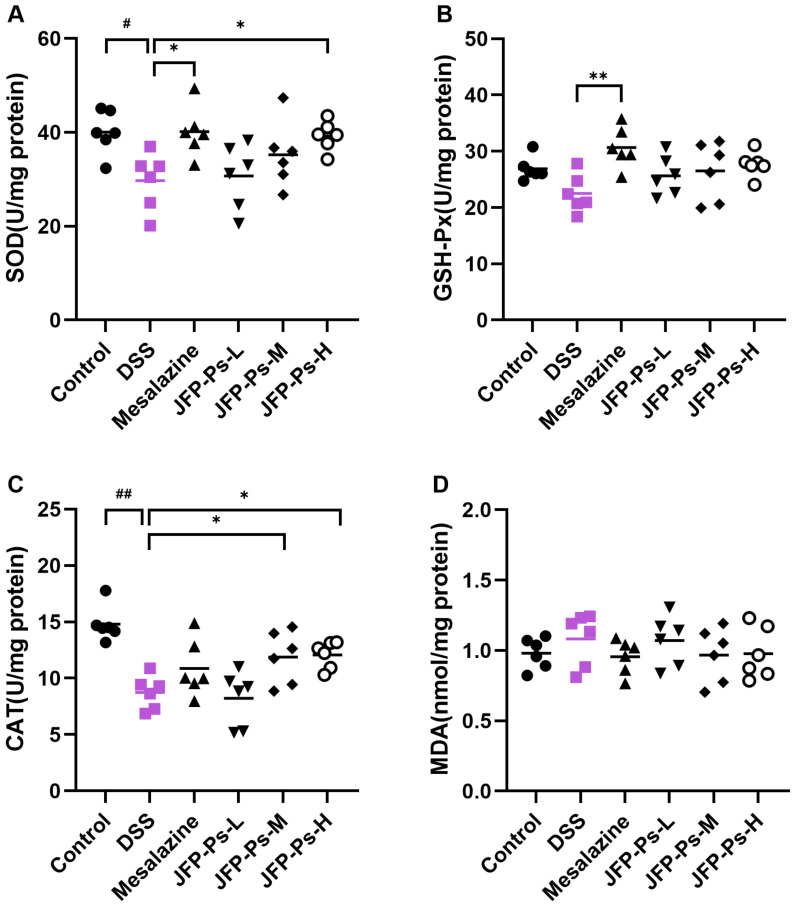
The protective effect of JFP-Ps against oxidative stress in the small intestines of DSS-induced enteritis rats. (**A**) SOD activity, (**B**) GSH-Px activity, (**C**) CAT activity, and (**D**) MDA level. # *p* < 0.05, ## *p* < 0.01 vs. Control group. * *p* < 0.05, ** *p* < 0.01 vs. DSS group.

The protective influence of JFP-Ps against oxidative stress damage in the small intestine was evaluated. As shown in Figure 2, DSS decreased the activities of SOD, GSH-Px, and CAT compared with the control group. However, JFP-Ps and mesalazine increased the activities of antioxidant enzymes in the small intestine compared to the DSS treatment group. Notably, treatments with JFP-Ps-M and JFP-Ps-H increased the activities of SOD and CAT significantly (*p* < 0.05). JFP-Ps treatment also decreased the content of MDA in the small intestines of rats with DSS-induced enteritis.

### 2.3. JFP-Ps Improved Inflammatory Cytokine Homeostasis in the Intestine

As shown in Table 1, the levels of pro-inflammatory cytokines (TNF-α, IL-1β, IL-6, and IFN-γ) were significantly increased in the small intestines of the DSS-treated rats (*p* < 0.05). However, JFP-Ps and mesalazine inhibited the DSS-induced increase in TNF-α, IL-1β, IL-6, and IFN-γ contents. Compared to the control group, the level of anti-inflammatory cytokine IL-10 was diminished in the small intestines of the DSS-treated rats, whereas JFP-Ps and mesalazine significantly increased the concentration of IL-10 (*p* < 0.05). These results suggested that JFP-Ps mitigated intestinal inflammation in the DSS-induced rats by modulating the secretion of pro- and anti-inflammatory cytokines.

### 2.4. JFP-Ps Decreased the Expression of the Genes Associated with Inflammation

**Figure 3:**
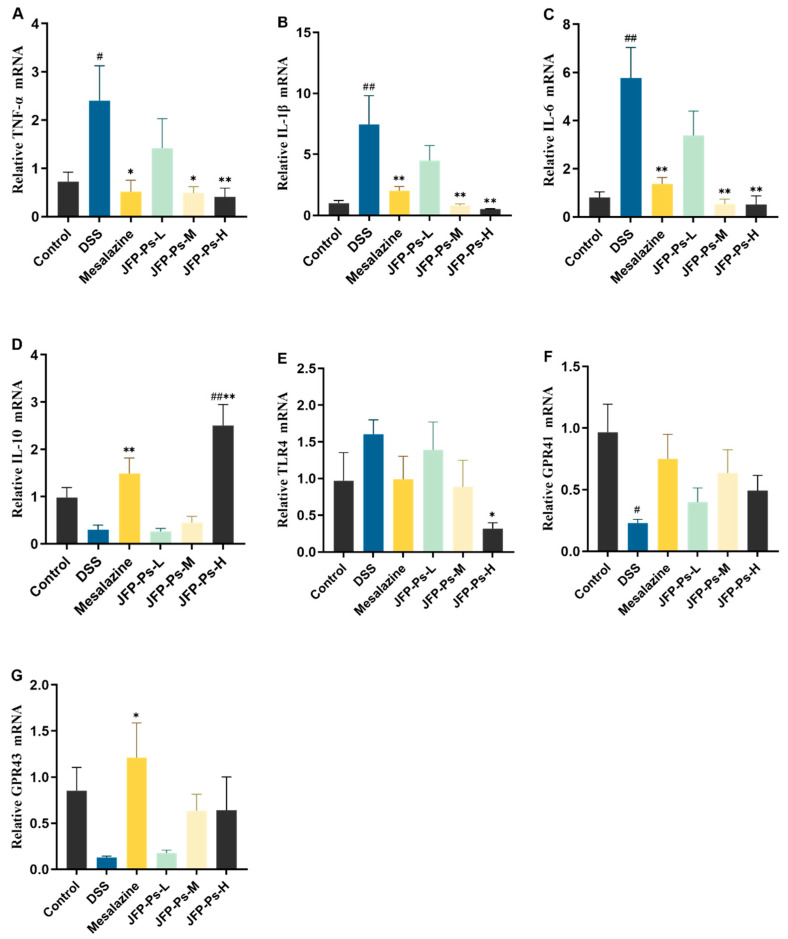
mRNA expression levels of (**A**) TNF-α, (**B**) IL-1β, (**C**) IL-6, (**D**) IL-10, (**E**) TLR4, (**F**) GPR41, and (**G**) GPR43 in the small intestine. # *p* < 0.05, ## *p* < 0.01 vs. Control group. * *p* < 0.05, ** *p* < 0.01 vs. DSS group.

RT-qPCR results (Figure 3) showed that DSS induced a significant increase in the mRNA expression of the pro-inflammatory cytokines (TNF-α, IL-1β, and IL-6) (*p* < 0.05). However, the mRNA expression of pro-inflammatory cytokines was significantly attenuated by JFP-Ps and mesalazine compared with the DSS group (*p* < 0.05). The IL-10 mRNA expression was enhanced in the mesalazine and JFP-Ps-H groups. The TLR4 mRNA expression was elevated in the small intestines of the DSS-induced rats, but suppressed by mesalazine and JFP-Ps. The mRNA expression levels of short-chain fatty acids (SCFAs) receptors GPR41 and GPR43 were diminished in the DSS-induced rats. However, mesalazine and JFP-Ps significantly increased the GPR41 and GPR43 mRNA expression (*p* < 0.05). These results suggested that JFP-Ps could modulate intestinal inflammatory responses by regulating the expression of the genes related to inflammation in the intestine and promoting the expression of SCFAs receptors.

### 2.5. JFP-Ps Enhanced the Expression of the Tight Junction Protein in the Small Intestine

**Figure 4:**
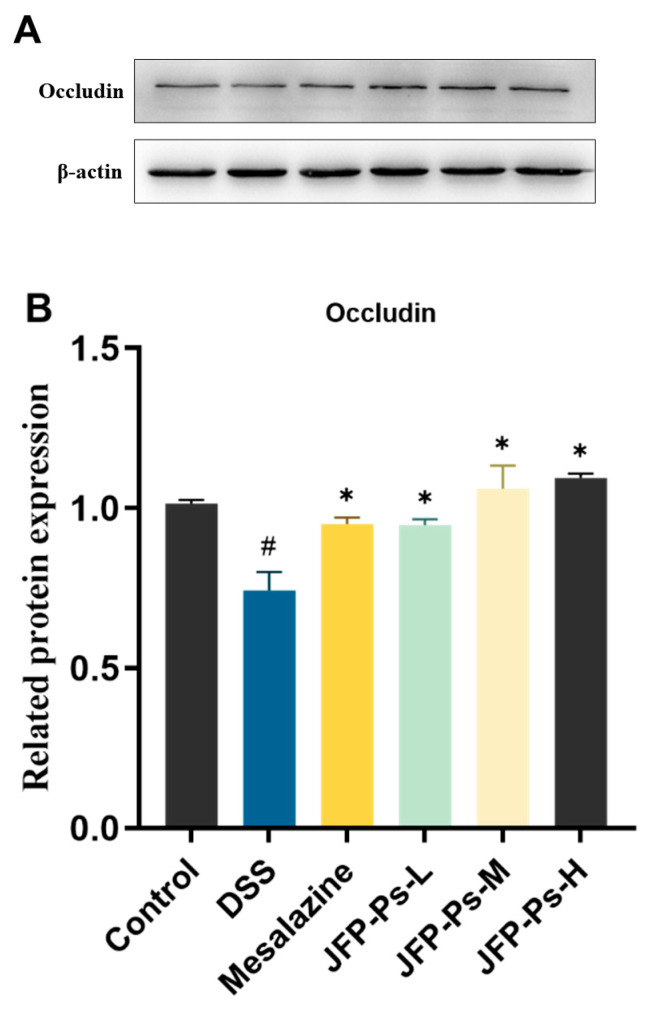
The expression of occludin in the small intestines. (**A**) Western blot analysis of occludin; (**B**) related protein expression of occludin. # *p* < 0.05 vs. Control group. * *p* < 0.05 vs. DSS group.

The impacts of JFP-Ps on the barrier function of the small intestine in the DSS-treated rats were investigated by examining the expression of the tight junction protein. Western blot analysis (Figure 4) showed a significant decrease in the protein expression level of occludin in the small intestines of the DSS-treated rats (*p* < 0.05). JFP-Ps significantly increased the protein expression of occludin (*p* < 0.05). These results indicated that JFP-Ps could enhance the mechanical barrier function of the small intestine by up-regulating the tight junction protein.

### 2.6. JFP-Ps Modulated the TLR4/MAPK Signaling Pathway in the Rats’ Small Intestines

**Figure 5:**
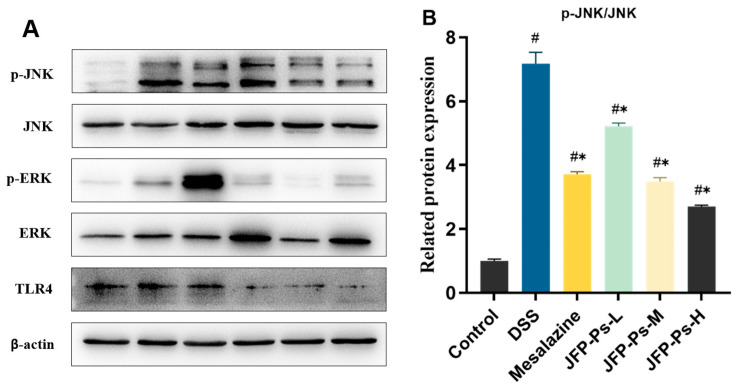

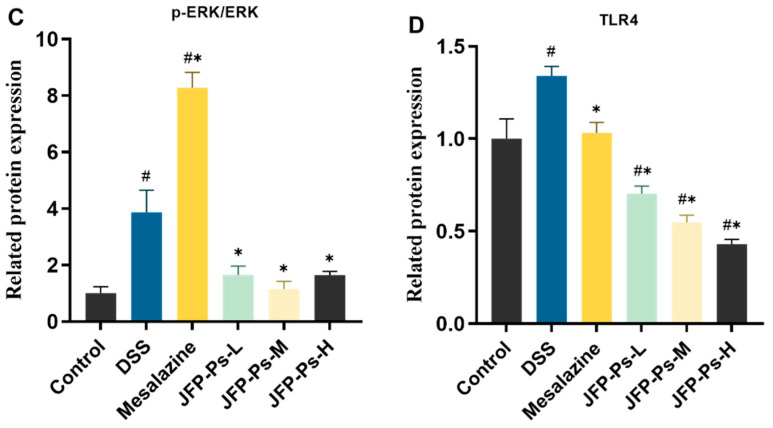
Expression of the proteins related to inflammatory pathways in the small intestine. (**A**) Phosphorylation levels of ERK and JNK and expression levels of TLR4 were analyzed with Western blotting. (**B**) p-JNK/JNK, (**C**) p-EKK/ERK, and (**D**) TLR4 expression. # *p* < 0.05 vs. Control group. * *p* < 0.05 vs. DSS group.

The anti-inflammatory mechanism of JFP-Ps in the small intestinal mucosal barrier is shown in Figure 5. The phosphorylation levels of JNK and ERK, and the expression of TLR4 in the DSS-treated rats, were notably higher than those in normal rats (*p* < 0.05). Compared to the DSS group, JFP-Ps substantially diminished the expression of TLR4 (*p* < 0.05) and curtailed the hyperphosphorylation of JNK and ERK. These results suggest that JFP-Ps could exert anti-inflammatory activity by inhibiting the TLR4/MAPK signaling pathway and safeguarding the intestinal mucosal barrier.

## 3. Discussion

Intestines are the largest organ for digestion and absorption in the body and possess crucial roles in human health. They are primarily composed of mucous membranes, which are involved in the digestion and assimilation of nutrients, and are the region with the most exposure to environmental factors [20]. As the body’s largest immunological organ, the intestines are pivotal in sustaining homeostasis and regulating immune function. However, the gastrointestinal tract is frequently affected by diseases such as IBD or chronic infections caused by immune deficiencies [21]. It has been reported that phytochemicals in the diet may protect the body from diseases by regulating intestinal epithelial barrier function [2]. This research suggested that JFP-Ps may safeguard intestinal function and health by regulating the inflammatory response and bolstering the intestinal barrier function.

The height of the intestinal villi, unique to the small intestine, is correlated with the contact area between the small intestine and nutrients. The crypt, a tubular gland formed in the lamina propria, can partially reflect the renewal status of epithelial cells based on its depth [6]. The current study established a model of DSS-induced intestinal inflammation in rats. Notable histological abnormalities were observed in the inflamed small intestines of the rats, characterized by atrophy of the intestinal villi and loss of epithelial cells. JFP-Ps ameliorated the histological damage and increased the height of small intestinal villi, suggesting that JFP-Ps may repair intestinal structural damage and alleviate the intestinal inflammatory response.

The intestinal mucosa serves as the body’s primary defense against the invasion of bacterial toxins and other exogenous pathogens, primarily comprising intestinal epithelial cells and tight junction proteins [22,23]. Occludin, an essential tight junction protein, plays a crucial role in maintaining intestinal barrier function and is closely related to the body’s antioxidant and anti-inflammatory capabilities [7,24]. Cui et al. [25] found that *Scutellaria baicalensis* Georgi polysaccharide improved the intestinal barrier by enhancing the expression of tight junction proteins, such as occludin. In this study, JFP-Ps notably augmented the expression of occludin, implying that JFP-Ps may mitigate intestinal mucosal damage and reinforce the mechanical barrier function of the small intestine.

Oxidative stress is closely associated with the inflammatory response, with episodes of intestinal inflammation potentially leading to an increased release of pro-inflammatory cytokines and chemokines. These can readily cause epithelial cell damage and result in the destruction of the intestinal mechanical barrier [26,27]. The antioxidant enzymes, SOD, GSH-Px, and CAT, are vital for the body to counteract oxidative stress damage and eliminate harmful oxidative metabolites produced by oxidative stress [28,29]. The level of MDA, a potent toxic product of lipid peroxidation, can serve as an indicator of cellular damage and excessive oxidative stress, reflecting the extent of tissue damage [30]. Lu et al. [31] reported that Iljinskaja polysaccharide and Chinese yam polysaccharide alleviated DSS-induced oxidative damage by regulating the activity of antioxidant enzymes. Our previous study found that JFP-Ps exhibited robust free radical scavenging ability [15]. In this study, JFP-Ps increased the activities of small intestinal SOD, GSH-Px, and CAT, and decreased MDA content. These results suggested that JFP-Ps may mitigate intestinal damage by enhancing antioxidant enzyme activity and modulating oxidative stress.

GPR41 and GPR43 are SCFAs receptors, which can influence the body’s metabolic and immune responses through various mechanisms, including regulating inflammatory responses and peptide hormone secretion [32,33]. SCFAs can bolster the intestinal barrier and inhibit pathogen invasion by activating the GPR41 and GPR43 receptors [34]. Lin et al. [35] reported that *Tetrastigma hemsleyanum* polysaccharides modulated immune signaling via activation of the SCFAs-GPR41/43 pathway, thereby preserving intestinal immune homeostasis. In the present study, JFP-Ps up-regulated the expression of GPR41 and GPR43 compared to the DSS-induced rats. These observations indicate that JFP-Ps may mitigate inflammation in the small intestine and augment the intestinal barrier function in rats through the SCFAs-GPR41/GPR43 pathway.

The immune system maintains homeostasis by regulating the secretion of inflammatory cytokines [36,37]. Overexpression of pro-inflammatory cytokines may cause inflammation in the intestinal mucosa [38]. TNF-α is a primary instigator of inflammatory injury, which stimulates the expression of IL-1β and IL-6, and further exacerbates the inflammatory response [28,39]. In addition, IFN-γ and anti-inflammatory cytokine IL-10 also play important roles in maintaining intestinal homeostasis. Guo et al. [40] reported that hawthorn polysaccharide mitigated intestinal inflammation by inhibiting the secretion of inflammatory cytokines, such as IL-1β, IL-6, and TNF-α. *Dictyophora indusiata* polysaccharide alleviated inflammatory injury by inhibiting the secretion of pro-inflammatory cytokines, such as TNF-α, IL-1β, IL-6, and IFN-γ, and by increasing the level of the anti-inflammatory factor IL-10 [41]. In this study, JFP-Ps reduced the expression of pro-inflammatory cytokines (TNF-α, IL-1β, IL-6, and IFN-γ) and increased IL-10 secretion. This observation implies that JFP-Ps may effectively mitigate the DSS-induced inflammatory response in the small intestine.

NF-κB and MAPK are two important signaling factors that regulate the inflammatory response. Activation of the NF-κB pathway can incite cytokine storms [42,43]. MAPKs are one of the most important pathways of the NF-κB signaling pathway [44,45]. This pathway is mainly activated by phosphorylated JNK and ERK. Moreover, polysaccharides have been reported to modulate the MAPK signaling pathway through TLR4, subsequently inducing cytokine expression [46]. Consistent with the results of previous studies on other natural plant polysaccharides [47], JFP-Ps significantly inhibited TLR4 expression and the phosphorylation of JNK and ERK. The findings indicated that JFP-Ps may mitigate inflammatory responses and preserve small intestinal barrier integrity through the modulation of the TLR4/MAPK signaling pathway.

## 4. Materials and Methods

### 4.1. Preparation of JFP-Ps

Jackfruits were obtained from the Xinglong Tropical Botanical Garden, Wanning, Hainan, China. JFP-Ps were extracted according to our previously reported method [15]. Jackfruits were collected at full maturity (14–16 weeks after flowering) and processed by dicing their flesh, followed by homogenization in a grinder. Subsequently, the homogenate was treated with 80% ethanol for a 24-hour period to remove non-target components. The dried material was dissolved in ultrapure water (material to liquid ratio, 1:30 mL/g) and subjected to extraction at 90 °C for 2.5 h in a water bath. The resultant aqueous extract was concentrated using a rotary evaporator at 55 °C under reduced pressure and subsequently filtered. Ethanol was added to precipitate the mixture at 4 °C overnight. The precipitate was redissolved in ultrapure water, and proteins were removed employing the Sevag method. Following a 72-hour dialysis, the solution underwent chromatographic separation and purification using a Sephacryl™ S-400 HR column (Sigma Chemical Co., St. Louis, MO, USA).

### 4.2. Materials and Reagents

Dextran sodium sulfate (DSS) was purchased from MP Biomedicals (Irvine, CA, USA). Assay kits for MDA, CAT, GSH-Px, and SOD were procured from Grace Biotechnology Co. (Suzhou, China). All of the primers were purchased from Sangon Biotech (Shanghai) Co., Ltd. (Shanghai, China). IL-1β, IL-6, TNF-α, IL-10, and IFN-γ ELISA kits were purchased from Shanghai Enzyme-linked Biotechnology Co., Ltd. (Shanghai, China). Polyclonal antibodies and secondary antibodies for occludin, JNK, P-JNK, ERK, P-ERK, and TLR-4 were from Proteintech Group, Inc. (Wuhan, China).

### 4.3. Experimental Animal Model

All the animal experiments were approved by the Animal Care and Use Committee of Hainan University, China. Forty-eight healthy male SD rats (180 ± 5 g) were purchased from Hunan SJA Laboratory Animal Co. (Changsha, China). They were maintained in individual cages under controlled temperatures (22–24 °C) and 12 h/12 h light/dark cycle conditions, with water and food provided ad libitum. After 1 week of adaptive feeding, the rats were randomly divided into 6 groups (8 rats per group): control group, DSS treatment group (DSS), mesalazine group, low, medium, and high dose JFP-Ps groups (JFP-Ps-L, JFP-Ps-M, and JFP-Ps-H). In the control group, rats were given distilled water, and rats in the DSS-induced group were given 3% DSS (*w*/*v*) solution periodically for 7 days. During the DSS treatment, the mesalazine group was given 10 mg/mL mesalazine solution by gavage daily, and the JFP-Ps-L (50 mg/kg JFP-Ps), JFP-Ps-M (100 mg/kg JFP-Ps), and JFP-Ps-H (200 mg/kg JFP-Ps) groups were orally treated with JFP-Ps daily.

### 4.4. Histological Analysis

The small intestine samples were preserved in neutral formalin for 24 h. Subsequently, these tissues were embedded in paraffin and sectioned at a 4 μm thickness. The sections were then mounted onto pre-treated slides and heated at 60 °C, then observed and analyzed using a light microscope after hematoxylin and eosin (H&E) staining.

### 4.5. Cytokine Analysis

A volume of 1.0 mL pre-cooled phosphate buffer solution (PBS) (*w*/*v*, 1/10) was added to the small intestinal tissue (100 mg) and then homogenized using a homogenizer. The supernatant was collected following centrifugation at 12,000× *g*, 4 °C for 20 min. All cytokine concentrations were quantified using ELISA kits (Shanghai Enzyme-linked Biotechnology Co., Ltd., Shanghai, China), adhering strictly to the instructions.

### 4.6. Antioxidant Activity Assays

A hundred milligrams of ileal tissue were mixed with 1 mL of the extract (*w*/*v*, 1:10), followed by a homogenization procedure using a homogenizer. After centrifugation at 12,000× *g*, 4 °C for 15 min, the supernatant was segregated. All antioxidant enzyme activities and MDA content were quantified by employing assay kits in strict adherence to the manufacturer’s instructions.

### 4.7. mRNA Quantification

The TriQuick reagent (Beyotime, Beijing, China) was used to extract total RNA from small intestinal tissue. The absorbance ratio at 260 and 280 nm was measured to quantify the purity and concentration of the retrieved RNA, employing a Thermo Scientific^TM^ NanoDrop^TM^ 2000C spectrophotometer (Waltham, MA, USA). The BeyoRTTM III First-Strand cDNA Synthesis Kit (Beyotime, Shanghai, China) was used to perform reverse transcription on the obtained RNA. The relative gene expression was subsequently measured with the CFX Connect real-qPCR system (BioRad, Hercules, CA, USA) and the SuperReal Preix Plus kit (SYBR Green) (Tiangen, Beijing, China). β-actin was used as a housekeeping gene and the data are expressed as relative values determined using the comparative threshold cycle (Cq) method (2^−ΔΔCq^). All primers utilized in this research are detailed in Table 2.

### 4.8. Protein Quantification and Western Blotting

Total proteins were extracted from the small intestine according to Kanwal et al. [48], with slight modification. Briefly, 0.1 g of small intestine sample was mixed with RIPA lysate, protease inhibitors, and phosphatase inhibitors (Beyotime, Shanghai, China), and then homogenized and centrifuged (10,000× *g*, 4 °C, 15 min). The protein concentration in the supernatant was quantified using a BCA protein assay kit (Solarbio, Beijing, China). The protein sample (35 µg) was resuspended in a sample loading buffer (with DTT) and boiled for 8 min, then separated by sodium dodecyl sulphate–polyacrylamide gel electrophoresis (SDS-PAGE). Proteins in the gels were electrophoretically transferred to polyvinylidene difluoride (PVDF) membranes using a transfer buffer containing ethanol at 120 V (constant voltage). The membrane was then treated with a western blocking buffer containing 5% bovine serum albumin (BSA) at room temperature for 1 h, then washed three times with TBST for 5 min each. Subsequently, the membrane was incubated at 4 °C with specific antibodies overnight. After washing with TBST, the membrane was incubated with HRP-conjugated Affinipure Goat Anti-Rabbit IgG (H+L) at room temperature for 2 h. Proteins were detected via chemiluminescence, employing a Tanon 5200 Multi-Chemiluminescence Imaging System. The luminescence intensity was normalized to β-actin.

### 4.9. Statistical Analysis

Results are expressed as mean ± standard deviation (SD). Data were statistically analyzed with one-way ANOVA, followed by *t*-test using GraphPad Prism 9 and SPSS 26.0 software. *p* < 0.05 was considered statistically significant.

## 5. Conclusions

The current study demonstrated the potential of JFP-Ps to mitigate and prevent inflammation in the small intestines of DSS-induced rats. JFP-Ps may reduce inflammatory damage in the small intestine by suppressing inflammatory responses, augmenting antioxidant capacity, and strengthening intestinal barrier function. Specifically, JFP-Ps may alleviate inflammatory injury in the small intestine and maintain cytokine homeostasis by inhibiting the activation of the TLR4/MAPK pathway. The intervention of JFP-Ps increased the activities of oxidative stress-related enzymes and reduced the content of MDA in the rats’ small intestines. JFP-Ps elevated the expression of GPR41/GPR43 mRNA and bolstered the protein expression of occludin. The findings of our study offer a theoretical foundation for the development of JFP-Ps as a natural immune regulator for intestinal health.

## Figures and Tables

**Table 1:** Effect of JFP-Ps on the levels of inflammatory cytokines in the small intestine.

Group	TNF-α (pg/mL)	IL-1β (pg/mL)	IL-6 (pg/mL)	IL-10 (pg/mL)	IFN-γ (pg/mL)
Control	281.22 ± 12.33	99.95 ± 7.09	106.86 ± 7.54	50.27 ± 1.11	1364.65 ± 46.68
DSS	325.74 ± 10.71 ^#^	120.88 ± 5.01 ^#^	132.06 ± 3.11 ^#^	46.75 ± 1.47	1605.46 ± 46.63 ^#^
Mesalazine	306.69 ± 7.84	116.94 ± 2.41 ^#^	119.99 ± 5.36	54.18 ± 1.81 *	1478.45 ± 58.66
JFP-Ps-L	319.32 ± 10.15 ^#^	120.44 ± 3.80 ^#^	130.57 ± 3.99 ^#^	52.97 ± 2.05 *	1579.04 ± 46.11 ^#^
JFP-Ps-M	308.73 ± 2.76	117.31 ± 2.97 ^#^	121.06 ± 2.83	54.12 ± 2.04 *	1569.89 ± 26.93 ^#^
JFP-Ps-H	306.99 ± 10.67	111.03 ± 1.84 ^#^	118.63 ± 2.82	54.36 ± 0.51 *	1446.95 ± 55.45 *

^#^ *p* < 0.05 vs. Control group; * *p* < 0.05 vs. DSS group.

**Table 2:** The primer sequences for amplification in RT-qPCR.

Primer	Forward 5′-3′	Reverse 5′-3′
β-actin	TGTCACCAACTGGGACGATA	GGGGTGTTGAAGGTCTCAAA
TLR-4	GGTTGGCACTCTCACTTCCTCTTG	GTAAATGGTGGCAGGGCAGAGTC
IL-1β	AATCTCACAGCAGCATCTCGACAAG	TCCACGGGCAAGACATAGGTAGC
IL-10	GGCAGTGGAGCAGGTGAAGAATG	TGTCACGTAGGCTTCTATGCAGTTG
IL-6	ACTTCCAGCCAGTTGCCTTCTTG	TGGTCTGTTGTGGGTGGTATCCTC
TNF-α	AAAGGACACCATGAGCACGGAAAG	CGCCACGAGCAGGAATGAGAAG
GPR41	TCTGCTCCTCTTCCTGCCATTCC	CGTTCTATGCTCACCGTCATCAGG
GPR43	TGCACCATCGTCATCATCGTTCAG	ACCAGGCACAGCTCCAGTCG

## Data Availability

The data that support the findings of this study are available from the corresponding author upon reasonable request.

## References

[1] Turner J.R. (2009). Intestinal mucosal barrier function in health and disease. Nat. Rev. Immunol..

[2] Ganesan K., Quiles J.L., Daglia M., Xiao J., Xu B. (2021). Dietary phytochemicals modulate intestinal epithelial barrier dysfunction and autoimmune diseases. Food Front..

[3] Zhou C.-B., Zhou Y.-L., Fang J.-Y. (2021). Gut microbiota in cancer immune response and immunotherapy. Trends Cancer.

[4] Huo J., Wu Z., Sun W., Wang Z., Wu J., Huang M., Wang B., Sun B. (2022). Protective effects of natural polysaccharides on intestinal barrier injury: A review. J. Agric. Food Chem..

[5] Peron G., Hidalgo-Liberona N., González-Domínguez R., Garcia-Aloy M., Guglielmetti S., Bernardi S., Kirkup B., Kroon P.A., Cherubini A., Riso P. (2019). Exploring the molecular pathways behind the effects of nutrients and dietary polyphenols on gut microbiota and intestinal permeability: A perspective on the potential of metabolomics and future clinical applications. J. Agric. Food Chem..

[6] Fang Q.-Y., Chen S.-P., Wang J.-Q., Huang X.-J., Nie Q.-X., Phillips G.O., Cui S.W., Li Y.-J., Nie S.-P. (2021). Fractions from natural Cordyceps sinensis alleviated intestinal injury in cyclophosphamide-induced mice. Bioact. Carbohydr. Diet. Fibre.

[7] Zhuang S., Zhong J., Bian Y., Fan Y., Chen Q., Liu P., Liu Z. (2019). Rhein ameliorates lipopolysaccharide-induced intestinal barrier injury via modulation of Nrf2 and MAPKs. Life Sci..

[8] Yang W., Zhao P., Li X., Guo L., Gao W. (2022). The potential roles of natural plant polysaccharides in inflammatory bowel disease: A review. Carbohydr. Polym..

[9] Song Q., Wang Y., Huang L., Shen M., Yu Y., Yu Q., Chen Y., Xie J. (2021). Review of the relationships among polysaccharides, gut microbiota, and human health. Food Res. Int..

[10] Jia J., Zhang P., Zhang C., Jiang G., Zheng W., Song S., Ai C. (2021). Sulfated polysaccharides from pacific abalone attenuated DSS-induced acute and chronic ulcerative colitis in mice via regulating intestinal micro-ecology and the NF-κB pathway. Food Funct..

[11] Xie Y., Wang L., Sun H., Wang Y., Yang Z., Zhang G., Jiang S., Yang W. (2019). Polysaccharide from alfalfa activates RAW 264.7 macrophages through MAPK and NF-κB signaling pathways. Int. J. Biol. Macromol..

[12] Wang K., Zhang H., Han Q., Lan J., Chen G., Cao G., Yang C. (2020). Effects of astragalus and ginseng polysaccharides on growth performance, immune function and intestinal barrier in weaned piglets challenged with lipopolysaccharide. J. Anim. Physiol. Anim. Nutr..

[13] Zou T., Yang J., Guo X., He Q., Wang Z., You J. (2021). Dietary seaweed-derived polysaccharides improve growth performance of weaned pigs through maintaining intestinal barrier function and modulating gut microbial populations. J. Anim. Sci. Biotechnol..

[14] Feng X., Du C., Wang C.J.I.J.o.B.M. (2021). Structural characterization of polysaccharide from yellow sweet potato and ameliorates DSS-induced mice colitis by active GPR41/MEK/ERK 1/2 signaling pathway. Int. J. Biol. Macromol..

[15] Zhu K., Zhang Y., Nie S., Xu F., He S., Gong D., Wu G., Tan L. (2017). Physicochemical properties and in vitro antioxidant activities of polysaccharide from *Artocarpus heterophyllus* Lam. pulp. Carbohydr. Polym..

[16] Tan Y.-F., Li H.-L., Lai W.-Y., Zhang J.-Q. (2013). Crude dietary polysaccharide fraction isolated from jackfruit enhances immune system activity in mice. J. Med. Food.

[17] Wiater A., Paduch R., Trojnar S., Choma A., Pleszczyńska M., Adamczyk P., Pięt M., Próchniak K., Szczodrak J., Strawa J. (2020). The effect of water-soluble polysaccharide from jackfruit (*Artocarpus heterophyllus* Lam.) on human colon carcinoma cells cultured in vitro. Plants.

[18] Zeng S., Chen Y., Wei C., Tan L., Li C., Zhang Y., Xu F., Zhu K., Wu G., Cao J. (2022). Protective effects of polysaccharide from *Artocarpus heterophyllus* Lam.(jackfruit) pulp on non-alcoholic fatty liver disease in high-fat diet rats via PPAR and AMPK signaling pathways. J. Funct. Foods.

[19] Zhu K., Fan H., Zeng S., Nie S., Zhang Y., Tan L., Li C., Xu F., Liu Q., Wu G. (2021). Polysaccharide from *Artocarpus heterophyllus* Lam. (jackfruit) pulp modulates gut microbiota composition and improves short-chain fatty acids production. Food Chem..

[20] Bernardi S., Del Bo’ C., Marino M., Gargari G., Cherubini A., Andrés-Lacueva C., Hidalgo-Liberona N., Peron G., González-Dominguez R., Kroon P. (2019). Polyphenols and intestinal permeability: Rationale and future perspectives. J. Agric. Food Chem..

[21] Santaolalla R., Abreu M.T. (2012). Innate immunity in the small intestine. Curr. Opin. Gastroenterol..

[22] Pawłowska B., Sobieszczańska B.M. (2017). Intestinal epithelial barrier: The target for pathogenic *Escherichia coli*. Adv. Clin. Exp. Med. Off. Organ Wroc. Med. Univ..

[23] Wu Y., Tang L., Wang B., Sun Q., Zhao P., Li W. (2019). The role of autophagy in maintaining intestinal mucosal barrier. J. Cell. Physiol..

[24] Zeng S., Cao J., Chen Y., Li C., Wu G., Zhu K., Chen X., Xu F., Liu Q., Tan L. (2022). Polysaccharides from *Artocarpus heterophyllus* Lam.(jackfruit) pulp improves intestinal barrier functions of high fat diet-induced obese rats. Front. Nutr..

[25] Cui L., Guan X., Ding W., Luo Y., Wang W., Bu W., Song J., Tan X., Sun E., Ning Q. (2021). Scutellaria baicalensis Georgi polysaccharide ameliorates DSS-induced ulcerative colitis by improving intestinal barrier function and modulating gut microbiota. Int. J. Biol. Macromol..

[26] Chen Y., Yang B., Ross R.P., Jin Y., Stanton C., Zhao J., Zhang H., Chen W. (2019). Orally administered CLA ameliorates DSS-induced colitis in mice via intestinal barrier improvement, oxidative stress reduction, and inflammatory cytokine and gut microbiota modulation. J. Agric. Food Chem..

[27] Lugrin J., Rosenblatt-Velin N., Parapanov R., Liaudet L. (2014). The role of oxidative stress during inflammatory processes. Biol. Chem..

[28] Chen S., Wu X., Tang S., Yin J., Song Z., He X., Yin Y. (2021). Eugenol alleviates dextran sulfate sodium-induced colitis independent of intestinal microbiota in mice. J. Agric. Food Chem..

[29] Wang X.Y., Yin J.Y., Hu J.L., Nie S.P., Xie M.Y. (2022). Gastroprotective polysaccharide from natural sources: Review on structure, mechanism, and structure–activity relationship. Food Front..

[30] Mao G., Li Q., Deng C., Wang Y., Ding Y., Zhang W., Chen Y., Zhao T., Wei F., Yang L. (2018). The synergism and attenuation effect of Selenium (Se)-enriched Grifola frondosa (Se)-polysaccharide on 5-Fluorouracil (5-Fu) in Heps-bearing mice. Int. J. Biol. Macromol..

[31] Lu H., Shen M., Chen Y., Yu Q., Chen T., Xie J.J.F.R.I. (2023). Alleviative effects of natural plant polysaccharides against DSS-induced ulcerative colitis via inhibiting inflammation and modulating gut microbiota. Food Res. Int..

[32] Kimura I., Ichimura A., Ohue-Kitano R., Igarashi M. (2020). Free fatty acid receptors in health and disease. Physiol. Rev..

[33] Kim M.H., Kang S.G., Park J.H., Yanagisawa M., Kim C.H. (2013). Short-chain fatty acids activate GPR41 and GPR43 on intestinal epithelial cells to promote inflammatory responses in mice. Gastroenterology.

[34] Kim M., Friesen L., Park J., Kim H.M., Kim C.H. (2018). Microbial metabolites, short-chain fatty acids, restrain tissue bacterial load, chronic inflammation, and associated cancer in the colon of mice. Eur. J. Immunol..

[35] Lin Y., Lv Y., Mao Z., Chen X., Chen Y., Zhu B., Yu Y., Ding Z., Zhou F.J.I.J.o.B.M. (2023). Polysaccharides from Tetrastigma Hemsleyanum Diels et Gilg ameliorated inflammatory bowel disease by rebuilding the intestinal mucosal barrier and inhibiting inflammation through the SCFA-GPR41/43 signaling pathway. Int. J. Biol. Macromol..

[36] Sanchez-Muñoz F., Dominguez-Lopez A., Yamamoto-Furusho J.K. (2008). Role of cytokines in inflammatory bowel disease. World J. Gastroenterol. WJG.

[37] Li F., Han Y., Cai X., Gu M., Sun J., Qi C., Goulette T., Song M., Li Z., Xiao H. (2020). Dietary resveratrol attenuated colitis and modulated gut microbiota in dextran sulfate sodium-treated mice. Food Funct..

[38] Li L., Qiu N., Meng Y., Wang C., Mine Y., Keast R., Guyonnet V. (2023). Preserved egg white alleviates DSS-induced colitis in mice through the reduction of oxidative stress, modulation of infl ammatory cytokines, NF-κB, MAPK and gut microbiota composition. Food Sci. Hum. Wellness.

[39] He L.-X., Wang J.-B., Sun B., Zhao J., Li L., Xu T., Li H., Sun J.-Q., Ren J., Liu R. (2017). Suppression of TNF-α and free radicals reduces systematic inflammatory and metabolic disorders: Radioprotective effects of ginseng oligopeptides on intestinal barrier function and antioxidant defense. J. Nutr. Biochem..

[40] Guo C., Wang Y., Zhang S., Zhang X., Du Z., Li M., Ding K. (2021). Crataegus pinnatifida polysaccharide alleviates colitis via modulation of gut microbiota and SCFAs metabolism. Int. J. Biol. Macromol..

[41] Kanwal S., Joseph T.P., Aliya S., Song S., Saleem M.Z., Nisar M.A., Wang Y., Meyiah A., Ma Y., Xin Y. (2020). Attenuation of DSS induced colitis by Dictyophora indusiata polysaccharide (DIP) via modulation of gut microbiota and inflammatory related signaling pathways. J. Funct. Foods.

[42] Gao W., Wang C., Yu L., Sheng T., Wu Z., Wang X., Zhang D., Lin Y., Gong Y. (2019). Chlorogenic acid attenuates dextran sodium sulfate-induced ulcerative colitis in mice through MAPK/ERK/JNK pathway. BioMed Res. Int..

[43] Cao H., Liu J., Shen P., Cai J., Han Y., Zhu K., Fu Y., Zhang N., Zhang Z., Cao Y. (2018). Protective effect of naringin on DSS-induced ulcerative colitis in mice. J. Agric. Food Chem..

[44] Coskun M., Olsen J., Seidelin J.B., Nielsen O.H. (2011). MAP kinases in inflammatory bowel disease. Clin. Chim. Acta.

[45] Xu D., Zhuang L., Gao S., Ma H., Cheng J., Liu J., Liu D., Fu S., Hu G. (2022). Orally Administered Ginkgolide C Attenuates DSS-Induced Colitis by Maintaining Gut Barrier Integrity, Inhibiting Inflammatory Responses, and Regulating Intestinal Flora. J. Agric. Food Chem..

[46] Barbosa J.R., dos Santos Freitas M.M., da Silva Martins L.H., de Carvalho Junior R.N. (2020). Polysaccharides of mushroom *Pleurotus* spp.: New extraction techniques, biological activities and development of new technologies. Carbohydr. Polym..

[47] Lu H., Shen M., Chen T., Yu Y., Chen Y., Yu Q., Chen X., Xie J. (2022). Mesona chinensis Benth Polysaccharides Alleviate DSS-Induced Ulcerative Colitis via Inhibiting of TLR4/MAPK/NF-κB Signaling Pathways and Modulating Intestinal Microbiota. Mol. Nutr. Food Res..

[48] Kanwal S., Joseph T.P., Owusu L., Xiaomeng R., Meiqi L., Yi X. (2018). A polysaccharide isolated from Dictyophora indusiata promotes recovery from antibiotic-driven intestinal dysbiosis and improves gut epithelial barrier function in a mouse model. Nutrients.

